# A biflavonoid‐rich extract from *Selaginella moellendorffii* Hieron. induces apoptosis via STAT3 and Akt/NF‐κB signalling pathways in laryngeal carcinoma

**DOI:** 10.1111/jcmm.15812

**Published:** 2020-09-01

**Authors:** Huiqi Huang, Ji Hao, Kejian Pang, Yibing Lv, Dingrong Wan, Chaoqun Wu, Yuanren Ma, Xinzhou Yang, Wei K. Zhang

**Affiliations:** ^1^ School of Pharmaceutical Sciences South‐Central University for Nationalities Wuhan China; ^2^ Hotian Uygur Pharmaceutical Co., Ltd Hotian China

**Keywords:** Akt/NF‐κB, apoptosis, biflavonoid‐rich extract, laryngeal carcinoma, *Selaginella moellendorffii* Hieron., STAT3

## Abstract

*Selaginella moellendorffii* Hieron. (SM), a perennial evergreen plant, has been used in the treatment of acute infectious hepatitis, thoracic and hypochondriac lumbar contusions, systemic oedema and thrombocytopaenia. However, the role of a biflavonoid‐rich extract from SM (SM‐BFRE) in anti‐larynx cancer has rarely been reported. In this study, the in vitro and in vivo anti‐laryngeal cancer activity and potential mechanisms of SM‐BFRE were investigated. An off‐line semipreparative liquid chromatography‐nuclear magnetic resonance protocol was carried out to determine six biflavonoids from SM‐BFRE. In vitro, MTT assay revealed that SM‐BFRE inhibited the proliferation of laryngeal carcinoma cells. A wound healing assay indicated that SM‐BFRE suppressed the migration of laryngeal cancer cells. Hoechst 33 258 and Annexin V‐FITC/PI double staining assays were performed and verified that SM‐BFRE induced apoptosis in laryngeal carcinoma cells. The Hep‐2 bearing nude mouse model confirmed that SM‐BFRE also exhibited anticancer effect in vivo. In addition, Western blot analysis demonstrated that SM‐BFRE exerted its anti‐laryngeal cancer effect by activating the mitochondrial apoptotic pathway and inhibiting STAT3 and Akt/NF‐κB signalling pathways. All results suggested that SM‐BFRE could be considered as a potential chemotherapeutic drug for laryngeal cancer.

## 1 INTRODUCTION

Laryngeal cancer is a common invasive malignant tumour of the head and neck, and an important cause of morbidity and mortality worldwide.^1^ It accounts for 5.7% to 7.6% of systemic malignant tumours.^2^ More than 150,000 new cases of laryngeal cancer are diagnosed each year,^3^ and its incidence is increasing.^4^ Current treatment methods mainly include surgery, chemotherapy, radiation therapy, and biological therapy, and sometimes a combination of these.^5^ Although these comprehensive treatments have improved the survival rate of patients, 30%‐40% of them still die from tumour recurrence or metastasis.^6^ Therefore, to improve the survival ratio of patients and alleviate the toxic side effects, finding excellent anticancer drugs and designing a more comprehensive treatment plan are of utmost priority.

Numerous studies have shown that natural products are useful in the treatment of cancer and constitute an important area of cancer drug research.^7^ For example, paclitaxel is one of the most famous cancer drugs.^8^ Approved by the FDA, it is believed to be useful in the treatment of Kaposi's sarcoma, as well as lung, breast and ovarian carcinomas.^9^ It has also been reported that camptothecin has a significant inhibitory effect on leukaemia.^10^ Several natural products play a vital role in the prevention of cancer and hardly exhibit obvious side effects.^11^ Therefore, it might be feasible to combat the human laryngeal cancer using natural products.

*Selaginella moellendorffii* Hieron. (SM) is the dry whole herb of Selaginella Spring, Selaginellaceae. Recent studies have shown that biflavonoids, the main chemical constituents of Selaginella, exhibit anti‐tumour activity.^12^, ^13^ However, so far, the pharmacological activity and mechanism of action of SM‐biflavonoids on laryngeal carcinoma cells have rarely been reported. In the present study, a biflavonoid‐rich extract from *Selaginella moellendorffii* Hieron. (SM‐BFRE) was prepared and purified by repeated column chromatography. The activity of SM‐BFRE on two kinds of laryngeal carcinoma cell lines (Hep‐2 and TU212) was examined in vitro and in vivo, and initial explorations of the potential mechanisms of action of SM‐BFRE against laryngeal cancer were outlined.

## 2 MATERIALS AND METHODS

### 2.1 Chemicals and reagents

High‐performance liquid chromatography (HPLC)‐grade solvents were used for chromatography, and all the other chemicals were of analytical‐reagent grade. HPLC‐grade acetonitrile (MeCN) and methanol were purchased from Anhui Tedia High Purity Solvents Co., Ltd. (Susong City, Anhui, China). Dulbecco's modified Eagle medium (DMEM) was purchased from Sigma‐Aldrich (St. Louis, MO, USA). Foetal bovine serum (FBS) and antibiotics (100 U/mL penicillin and 100 μg/mL streptomycin) were obtained from Hyclone (Logan, UT, USA). Antibodies of β‐actin, MMP‐9, Bcl‐2, Bax, caspase‐9, caspase‐3, PARP, STAT3, p‐STAT3 (Tyr705), Akt, p‐Akt (Ser473) and NF‐κB p65 were obtained from Cell Signaling Technology (Danvers, MA, USA). The corresponding secondary antibody was obtained from Abcam (Cambridge, MA, USA).

### 2.2 Plant material

The whole parts of SM were purchased from the herb market of Nanning City, Guangxi Zhuang Autonomous Region, China, in August 2018. A voucher specimen (SC0064) is deposited in School of Pharmaceutical Sciences, South‐Central University for Nationalities (SCUN), Wuhan, China. The specimen of SM could be found in Figure S1.

### 2.3 Preparation and Chemical elucidation of SM‐BFRE

Dried whole plant of SM (500 g) was frozen with liquid nitrogen and ground with a YB‐2000A high speed pulverizer (Yongkang Sufeng Industry and Trade Co., Ltd, Yongkang City, China). The crushed residue was filtered through 200 mesh sieve, and the uniform powder was then extracted sequentially by maceration with 95% EtOH four times (2.5 L each) at room temperature. The solvent was evaporated under reduced pressure to obtain a crude extract (61.5 g). The extract was suspended in warm water and then partitioned successively with petroleum ether (PE), ethyl acetate (EtOAc) and n‐butyl alcohol (n‐BuOH) to afford PE fraction (4.3 g), EtOAc fraction (9.4 g) and n‐BuOH fraction (23.7 g), respectively. The ethyl acetate extract (8 g) was subjected to D101 macroporous resin column chromatography (200 g, Sinopharm Chemical Reagent Co., Ltd., Shanghai, China) eluted with 10, 30, 50, 70, 80 and 95% aq. ethanol in a gradient manner to give 142 eluents. The different eluents were combined to seven fractions (Frs. A‐G) according to the HPLC analysis results. Fr E showed the strongest cytotoxicity against the laryngeal carcinoma cell line Hep‐2 as the purified biflavonoid‐rich extract (SM‐BFRE, 3.5 g).

The chemical profiling of SM‐BFRE was performed by HPLC‐PDA method as previously described.^14^ The gradient elution was optimized for analysis with a Phenomenex Gemini‐NX C18 HPLC column (5 μm, 4.6 × 250 mm) (acetonitrile in water with 0.1% formic acid). The gradient program was as follows: 0‐13 minutes, 40%‐70% acetonitrile; 13‐13.05 minutes, 70%‐90% acetonitrile; 13.05‐20 minutes, 90%‐95% acetonitrile; and 20‐25 minutes, 95% acetonitrile. The flow rate of the analysis was 1.0 mL/min. The separation of SM‐BFRE was performed by the procedure as previously described.^15^ 100 mg of SM‐BFRE was dissolved in 2.0 mL of methanol, and the solution was filtered. 100 μL of SM‐BFRE was subjected to a semipreparative HPLC with a Phenomenex Gemini C18 HPLC column (5 μm, 10 × 250 mm) (acetonitrile in water with 0.1% formic acid from 40% to 60% for 16 minutes, from 60% to 90% for next 0.05 minutes, from 90% to 95% for next 8.95 minutes, 95% acetonitrile holding for 5 minutes). The flow rate of the analysis was 4.0 mL/min. A total of 6 peak‐based fractions were collected manually, and 20 injections were repeated to yield compounds **1** (52.7 mg), **2** (7.8 mg), **3** (4.7 mg), **4** (2.3 mg), **5** (8.2 mg) and **6** (2.5 mg). Compounds **1**‐**6** were dissolved in DMSO‐*d*6 for NMR with the amount range of 8.5‐2.3 mg for NMR tests.

### 2.4 Cell culture

Human laryngeal carcinoma cell line Hep‐2 was purchased from the American Type Culture Collection (Manassas, VA, USA). Human laryngeal cancer cell TU212 and HBE cell (normal laryngeal epithelial cells originated from human) were obtained from the Shanghai Institute of Biochemistry and Cell Biology, Chinese Academy of Sciences (Shanghai, China). The cells were cultured in DMEM supplemented with 10% (v/v) FBS and 1% penicillin/streptomycin. All cells were maintained in 25 cm^2^ or 75 cm^2^ cell culture flasks and cultured in an incubator containing a humidified atmosphere with 5% CO_2_ at 37°C.

### 2.5 MTT assay

Hep‐2, TU212 and HBE cells in logarithmic growth phase were inoculated at 1 × 10^4^ cells/well in 96‐well plates and cultured until about 80% fusion was achieved. Then, cells were incubated with 0, 5, 10, 20, 50 and 100 μg/mL SM‐BFRE for 12, 24 and 48 h. Following the treatment time, 10 μL MTT (5 mg/mL) and 90 μL DMEM were added to each well. After incubating for 30 ‐ 60 minutes, the liquid in each well was removed and 150 μL DMSO was added. The absorbance was measured by a microplate reader (Bio‐Rad, CA, USA) at 562 nm. The inhibition rate was calculated by the following formula: inhibition rate (%) = [1 − (OD_sample_ − OD_blank_)/(OD_control_ − OD_blank_)] × 100. IC_50_ was calculated by the GraphPad Prism 5.0 Software.

### 2.6 Wound healing assay

Hep‐2 and TU212 cells in logarithmic phase were inoculated at 2 × 10^5^ cells/well in 6‐well plates and cultured until about 80% fusion was achieved. After treatment with 0, 5, 10 and 20 μg/mL SM‐BFRE, wounds were created by scratching the plate surface with a sterile 200 μL pipette tip. Next, under an inverted phase contrast microscope (Soptop ICX41, Ningbo, China), the scratch wound images were photographed by the camera at 20 × magnification following 0, 12 and 24 h of SM‐BFRE incubation. The measurements of the wound surface area were calculated by ImageJ software.

### 2.7 Morphology observation and Hoechst 33 258 staining

Laryngeal cancer cells in logarithmic phase were inoculated at 2 × 10^5^ cells/well in 6‐well plates and cultured until about 80% fusion was achieved. After exposure to 0, 10, 30 and 50 μg/mL SM‐BFRE for 24 h, the cellular morphological changes were photographed at 40 × magnification under an inverted phase contrast microscope (Soptop ICX41, Ningbo, China). Subsequently, the original medium was removed, and a cell fixing solution (glacial acetic acid: methanol = 1:3) was added to each well for 15 minutes. The cells were then stained with Hoechst 33 258 (Beyotime, Shanghai, China) for 15 minutes in the dark. The cellular morphology was observed under a fluorescence microscope (excitation wavelength 350 nm, emission wavelength 460 nm, Soptop ICX41, Ningbo, China) and photographed at 40 × magnification.

### 2.8 Annexin V‐FITC/PI double staining assay

Laryngeal cancer cells in logarithmic phase were inoculated at 2 × 10^5^ cells/well in 6‐well plates and cultured until about 80% fusion was achieved. After incubation with 0, 10, 30 and 50 μg/mL SM‐BFRE for 24 hours, the cells were digested with trypsin without ethylenediaminetetraacetic acid (EDTA) and centrifuged to collect the suspended cells. Next, Annexin V‐FITC/PI staining was performed following the protocol described in the kit (BestBio, Shanghai, China). Immediately after staining, the proportion of apoptotic cells was measured by flow cytometry (Guava easyCyte, United States) and qualitatively observed with a fluorescence microscope. The percentage of apoptotic cells was analysed by FlowJo V10 software.

### 2.9 Protein preparation and Western blot analysis

Hep‐2 and TU212 cells in logarithmic phase were inoculated at 6 × 10^5^ cells/dish in 100 mm × 20 mm dishes and allowed to grow for 48 hours. After incubation with 0, 10, 30 and 50 μg/mL SM‐BFRE for 24 hours, the cells were collected by centrifugation and lysed in radio immunoprecipitation assay (RIPA) lysis buffer (Beyotime) to extract the proteins. The supernatant containing the protein was collected, and the concentration of the protein was measured by the bicinchoninic acid (BCA) kit (Beyotime). After denaturation, the protein was stored in a −80°C freezer until required. Protein extraction from the tumour tissues was performed following the same protocol. Equivalent amounts of the protein (40 µg) were separated by SDS‐PAGE gel electrophoresis and transferred to polyvinylidene difluoride (PVDF) membranes (Bio‐Rad). The membranes were blocked by 5% skim milk formulated with tris‐buffered saline with 0.5% Tween 20 (TBST) for 2 hours at room temperature. The membranes were then incubated with the primary antibody at 4°C overnight and incubated with the secondary antibody at room temperature for 1‐2 hours. The HRP ECL system (Bio‐Rad) was used to detect the protein band, and the grey value was calculated by Image Lab software.

### 2.10 Tumour‐bearing nude mouse models and in vivo treatment

Hep‐2 cells were inoculated to the right back of the nude mice (BALB/c, SPF grade, female, 4‐5 weeks old) at a concentration of 1 × 10^7^ per mouse. When the tumours grew to about 100 mm^3^, the mice were randomly divided into three groups (five mice in each group) and given an intraperitoneal injection (i.p.) of SM‐BFRE (0, 45 and 90 mg/kg/day) for 28 days. Tumour size was measured every 4 days. The length and width of transplanted tumours in mice were measured by a vernier calliper, and tumour volume was calculated by the following formula: Tumour volume = 0.5 × width^2^ × length. After 28 days, the transplanted tumours were removed for assessments. Athymic nude mice were purchased from the Beijing HFK Bioscience Co, Ltd (SCXK 2014‐0004). All the programs were in line with the requirements of the Animal Ethics Committee of SCUN (Approval Number: S08918121A) and were carried out in accordance with the Guide for the Care and Use of Laboratory Animals of the Ministry of Health, China.

### 2.11 Terminal deoxynucleotidyl transferase dUTP nick end labelling (TUNEL)

Tumours were embedded in paraffin and cut into 5 μm sections after pre‐treatment with cold saline. They were then fixed in a 10% formalin buffered neutral solution. After the sections were stained with the TUNEL assay kits (Roche Biotechnology, Basel, Switzerland), their photographs were taken at 200 × magnification. The brown‐yellow staining of the nucleus represented cell apoptosis.

### 2.12 Immunohistochemical examinations of transplanted tumour tissues (IHC)

Tumours were embedded in paraffin and cut into 5 μm sections. Their slices were then incubated with the desired primary antibody and visualized by corresponding secondary antibodies conjugated with horseradish peroxidase. After staining and sealing, photographs were taken at 200 × magnification. Positive staining was quantified using ImageJ software.

### 2.13 Statistical analysis

All data are shown as mean ± SD from at least three independent experiments. GraphPad Prism 5.0 software was used for all statistical analyses. Statistical differences were analysed by one‐way analysis of variance (ANOVA), and *P* values < .05 were considered significant (*P < .05, **P < .01 or ***P < .001).

## 3 RESULTS

### 3.1 Chemical characterization of SM‐BFRE

**FIGURE 1:**
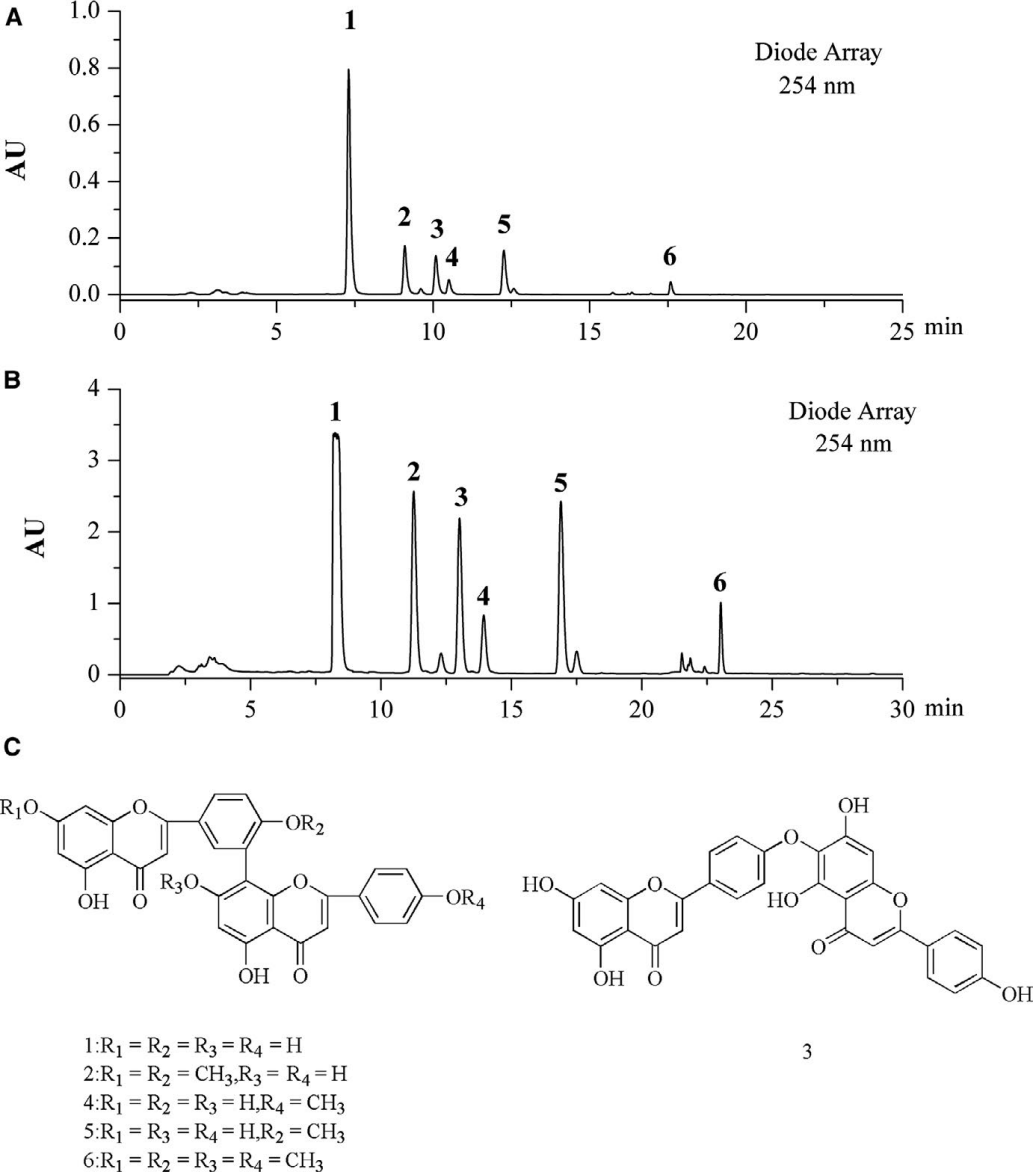
Chemical characterization of SM‐BFRE. (A) The LC‐PDA analysis of SM‐BFRE is shown at 254 nm with peak labelling corresponding to compounds 1‐6. (B) The optimized semipreparative HPLC separation of the SM‐BFRE is shown at 254 nm with peaks 1‐6 for NMR and further purified if required. (C) Structures of 6 compounds from SM‐BFRE

A HPLC–PDA method was carried out for chemical analysis of the main chemical constituents of SM‐BFRE (Figure 1A). A semipreparative HPLC chromatogram was used for large‐scale purification of the main chemical principles with the optimized gradient conditions (Figure 1B). Six peaks were collected, and 20 injections were repeated to give 6 pure compounds. The six compounds were elucidated as amentoflavone (**1**),^16^ ginkgetin (**2**),^16^ hinokiflavone (**3**),^17^ podocarpusflavone A (**4**),^18^ bilobetin (**5**) ^16^ and 4',4''',7,7''‐tetra‐O‐methylamentoflavone (**6**) ^19^ by comparison of their MS and NMR spectra along with published reference data (Figure 1C).

### 3.2 Effects of SM‐BFRE on proliferation of laryngeal carcinoma cells in vitro

**FIGURE 2:**
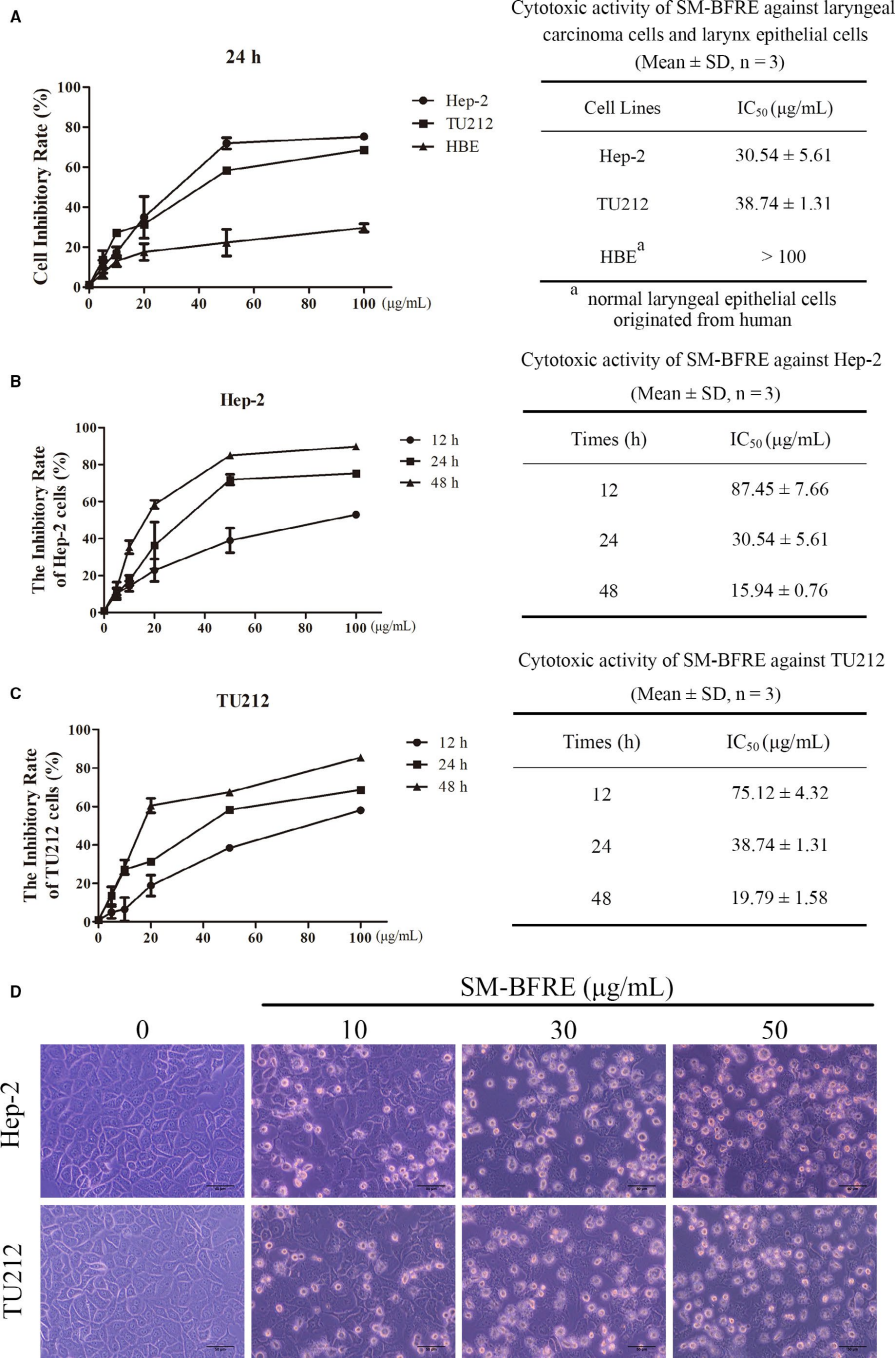
Effects of SM‐BFRE on proliferation of laryngeal carcinoma cells in vitro. (A) Cell viability and IC_50_ of Hep‐2, TU212 and HBE cells after SM‐BFRE incubation (0, 5, 10, 20, 50 and 100 μg/mL) for 24 h were detected by MTT assay. (B and C) Two laryngeal carcinoma cell lines were incubated, respectively, with SM‐BFRE (0, 5, 10, 20, 50 and 100 μg/mL) for 12, 24 and 48 h. The inhibitory rate and IC_50_ were detected by MTT assay. (D) Morphological changes of two laryngeal carcinoma cells after they were incubated with SM‐BFRE (0, 10, 30 and 50 μg/mL) for 24 h

The anti‐proliferative effects of SM‐BFRE in laryngeal carcinoma cells were assessed by MTT assay. It was observed that the IC_50_ of Hep‐2 and TU212 cells after 24 h of incubation with SM‐BFRE were 30.54 ± 5.61 μg/mL and 38.74 ± 1.31 μg/mL. At the same time, there was no obvious anti‐proliferative effect of SM‐BFRE on HBE cells at 24 h (IC_50_ > 100 μg/mL) (Figure 2A). In addition, SM‐BFRE inhibited laryngeal carcinoma cells in a dose‐ and time‐dependent manner (Figure 2B, C). Simultaneously, morphological observations revealed that SM‐BFRE treatment induced cell contraction, deformation and floating (Figure 2D). The cell viability and IC_50_ of two laryngeal cancer cells after treatment with 6 pure compounds for 12, 24 and 48 hours could be found in Figure S2. In order to verify that the activity of SM‐BFRE was mainly contributed by 6 major compounds and the minor compounds had almost no effect on the anti‐laryngeal cancer activity of the extract, we used a semipreparative HPLC to prepare the mixture of 6 major compounds and the mixture of minor compounds in SM‐BFRE. SM‐BFRE, the mixture of 6 major compounds, and the mixture of minor compounds were all diluted with DMEM to 100 μg/mL and their inhibitory rate on the two laryngeal cancer cells within 24 hours was tested by MTT assay. The results are shown in Table S1 in the Supplement Material. It showed that the inhibitory rate of the mixture of 6 major compounds on Hep‐2 and TU212 cells within 24 hours accounted for 95.17% and 93.92%, respectively, of SM‐BFRE under the same condition. The inhibitory rate of the mixture of minor compounds on Hep‐2 and TU212 cells was 3.24% and 5.26% of SM‐BFRE, respectively. It suggested that the activity of the extract was mainly contributed by 6 major compounds.

### 3.3 Effect of SM‐BFRE on the migration of laryngeal carcinoma cells

**FIGURE 3:**
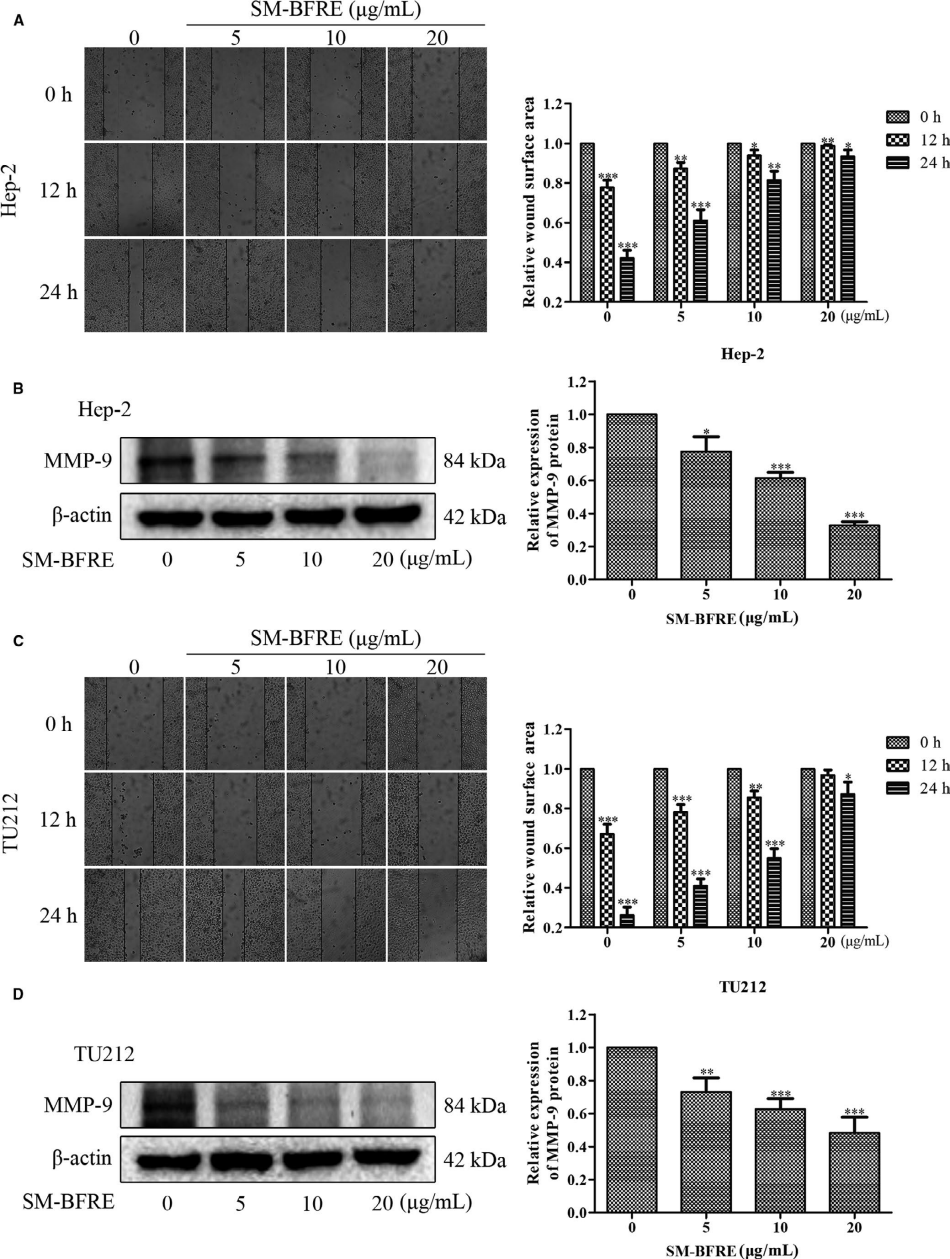
Effect of SM‐BFRE on the migration of laryngeal carcinoma cells. (A and C) Micrographs of two laryngeal carcinoma cells at 0, 12 and 24 h after treatment with SM‐BFRE (0, 5, 10 and 20 μg/mL), respectively, and relative wound surface area in wound healing assay was calculated by ImageJ software. (B and D) The expression of MMP‐9 protein was detected by Western blot assay, and the relative expression was calculated by Image Lab software. β‐actin was used as a control. *P < .05, **P < .01 or ***P < .001 compare to control (0 μg/mL served as control)

The effect of SM‐BFRE on laryngeal cancer cells was investigated through wound healing assay. When the SM‐BFRE concentration was more than 30 μg/mL, the proliferation of two laryngeal carcinoma cells was significantly inhibited. Therefore, SM‐BFRE concentrations of 0, 5, 10 and 20 μg/mL were applied. It could be clearly observed that as time progressed, the wound healed more. At the same time, with an increase in the dose, the inhibitory effect of SM‐BFRE on the migration of both laryngeal carcinoma cells also had gradually increased (Figure 3A, C). Matrix metalloproteinases (MMPs) play a major role in the metastasis and invasion of tumour cells.^20^ Among them, MMP‐9 plays a key role in promoting cell migration. The expression of MMP‐9 protein in two laryngeal carcinoma cells was detected by Western blot assay, and it was found that MMP‐9 expression decreased as the dose of SM‐BFRE was increased (Figure 3B, D

). This result indicated that SM‐BFRE had a significant inhibitory effect on the migration of laryngeal carcinoma cells.

### 3.4 Effect of SM‐BFRE on apoptosis of laryngeal carcinoma cells

**FIGURE 4:**
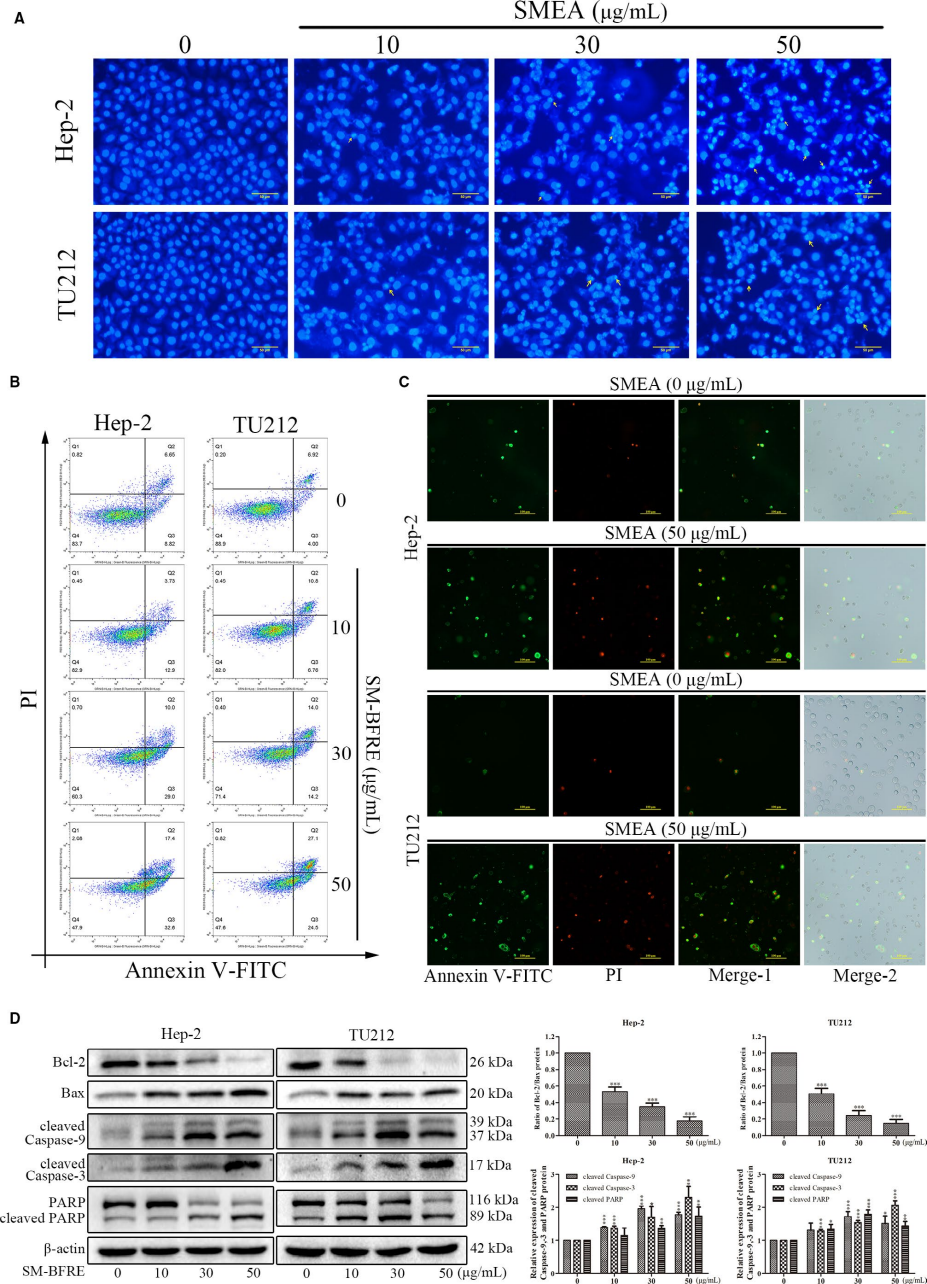
Effects of SM‐BFRE on apoptosis of laryngeal carcinoma cells. (A) Changes in two laryngeal carcinoma cells incubated with SM‐BFRE (0, 10, 30 and 50 μg/mL) for 24 h and stained with Hoechst 3328 under a fluorescence microscope. (B) Hep‐2 and TU212 cells were incubated with SM‐BFRE (0, 10, 30 and 50 μg/mL) for 24 h, cells were stained with Annexin V‐FITC and PI, and the proportion of apoptosis was detected by the flow cytometry. (C) Apoptosis of laryngeal carcinoma cells incubated with SM‐BFRE (0 and 50 μg/mL) was observed under a fluorescence microscope after detection by the flow cytometry. (D) The relative expression of Bcl‐2/Bax, cleaved caspase‐9 and caspase‐3 and PARP was detected by Western blot assay of two laryngeal carcinoma cells incubated with SM‐BFRE (0, 10, 30 and 50 μg/mL) for 24 h. β‐actin was used as a control. *P < .05, **P < .01 or ***P < .001 compare to control (0 μg/mL served as control)

Hoechst 33 258 staining was used to detect apoptosis in the laryngeal cancer cell lines. After staining, the chromatin in the nucleus of apoptotic cells condensed and bright apoptotic bodies were produced. Cells with these characteristics increased in number as the dosage was increased (Figure 4A). The results of Annexin V‐FITC/PI double staining assay were observed by the flow cytometry and a fluorescence microscope. Among the results detected by flow cytometry, Q2 + Q3 represented the proportion of apoptosis cells. Therefore, after 24 hours treatment with 0, 10, 30 and 50 μg/mL SM‐BFRE, the percentages of apoptotic cells were, respectively, 15.47%, 16.63%, 39.00% and 50.00% in Hep‐2 cells, and 10.92%, 17.56%, 28.20% and 51.60% in TU212 cells. This suggested that SM‐BFRE dose‐dependently induced apoptosis in laryngeal carcinoma cells (Figure 4B). Under a fluorescence microscope, the early apoptotic cells were stained with Annexin V‐FITC to give the cell membrane a green fluorescence, on the basis of which the late apoptotic cells were stained with propidium iodide (PI) to impart red fluorescence to the nucleus. The results indicated that the number of apoptotic cells was significantly increased after SM‐BFRE treatment (Figure 4C).

The regulation of SM‐BFRE on the mitochondrial apoptosis pathway of laryngeal carcinoma cells was detected by Western blot assay. The results indicated that with an increase in the SM‐BFRE concentration, the ratio of Bcl‐2/Bax was significantly decreased, while the expressions of cleaved caspase‐9 and caspase‐3 and PARP had gradually increased (Figure 4D). This suggested that SM‐BFRE might induce apoptosis in laryngeal carcinoma cells in vitro by activating the mitochondrial apoptotic pathway.

### 3.5 Inhibitory effect of SM‐BFRE on the growth of transplanted tumours and the effect on apoptosis in vivo

**FIGURE 5:**
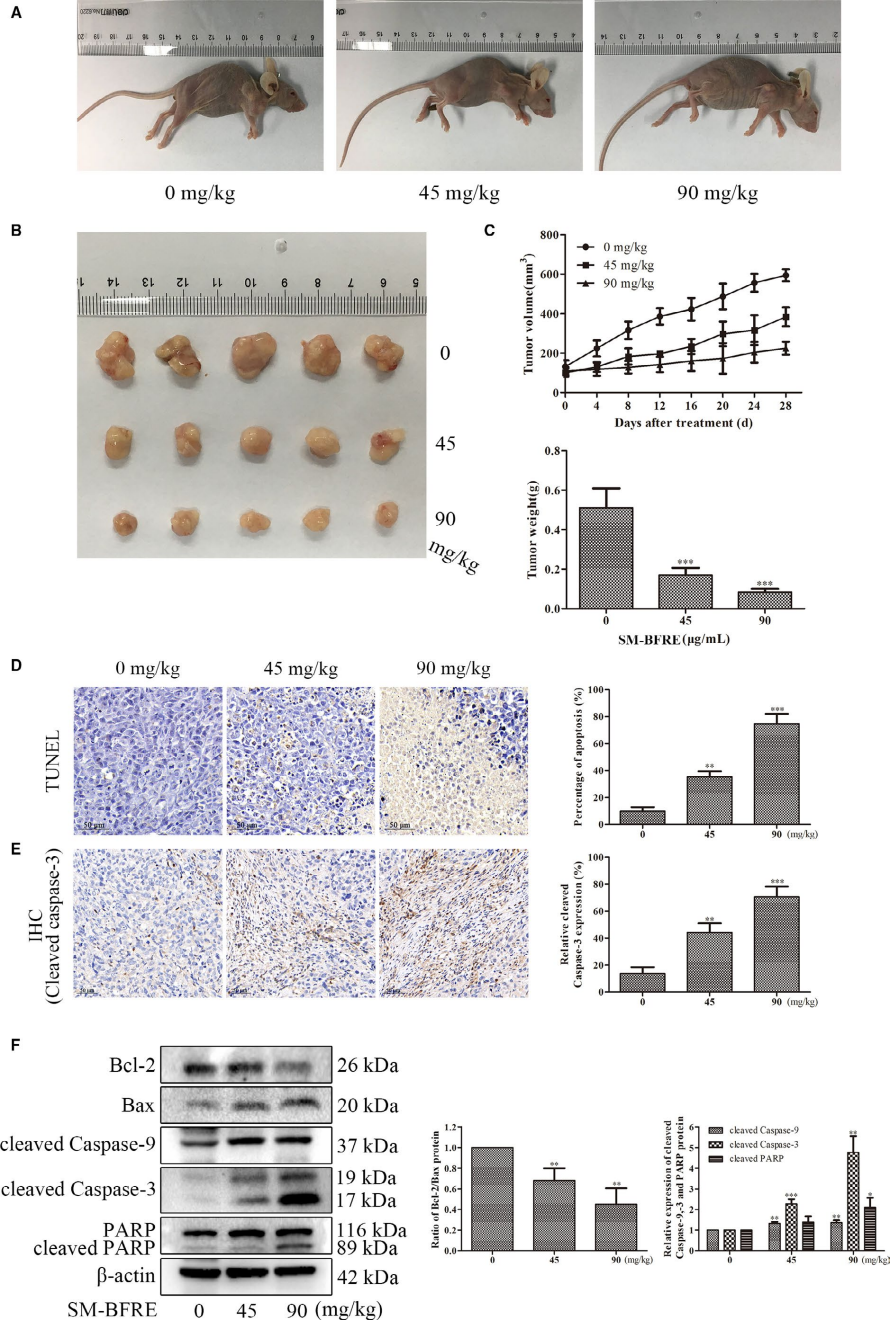
Inhibitory effect of SM‐BFRE on the growth of transplanted tumours and the effect on apoptosis in vivo. (A and B) After Hep‐2‐bearing mice were treated with SM‐BFRE (0, 45 and 90 mg/kg/day) for 4 weeks, the size of tumours in vivo was measured, and then tumours were removed. (C) The volume of the transplanted tumours was measured with a vernier calliper every 4 days during the experiment, and when the experiment was over, the tumours were removed and weighted. (D and E) Tumour sections were prepared and subjected to TUNEL and IHC staining, and the proportion of apoptotic cells was calculated. (F) Proteins were extracted from transplanted tumour tissues, and the relative expressions of Bcl‐2/Bax, cleaved caspase‐9 and caspase‐3 and PARP were detected by Western blot. β‐actin was used as a control. *P < .05, **P < .01 or ***P < .001 compare to control (0 μg/mL or 0 mg/kg served as control)

Hep‐2‐bearing nude mice were used as models to evaluate the role of SM‐BFRE in the prevention of laryngeal carcinoma in vivo. It was observed that SM‐BFRE dose‐dependently inhibited the proliferation of transplanted tumours in nude mice (Figure 5A‐C). The effect of SM‐BFRE on apoptosis in vivo was examined by TUNEL and immunohistochemical staining (IHC). The nuclei of the apoptotic cells were stained brown by TUNEL and IHC, showing that that as the dose of SM‐BFRE was increased, the number of apoptotic cells in the transplanted tumours also gradually increased (Figure 5D, E). The role of SM‐BFRE in promoting apoptosis of laryngeal cancer cells in vivo was further confirmed by Western blot analysis. It was found that the expression of relative proteins was consistent with the results obtained in vitro (Figure 5F). These results suggested that SM‐BFRE might also induce apoptosis in laryngeal carcinoma cells in vivo through the mitochondrial apoptotic pathway.

### 3.6 Regulation of STAT3 and Akt/NF‐κB signalling pathway by SM‐BFRE in vitro and in vivo

**FIGURE 6:**
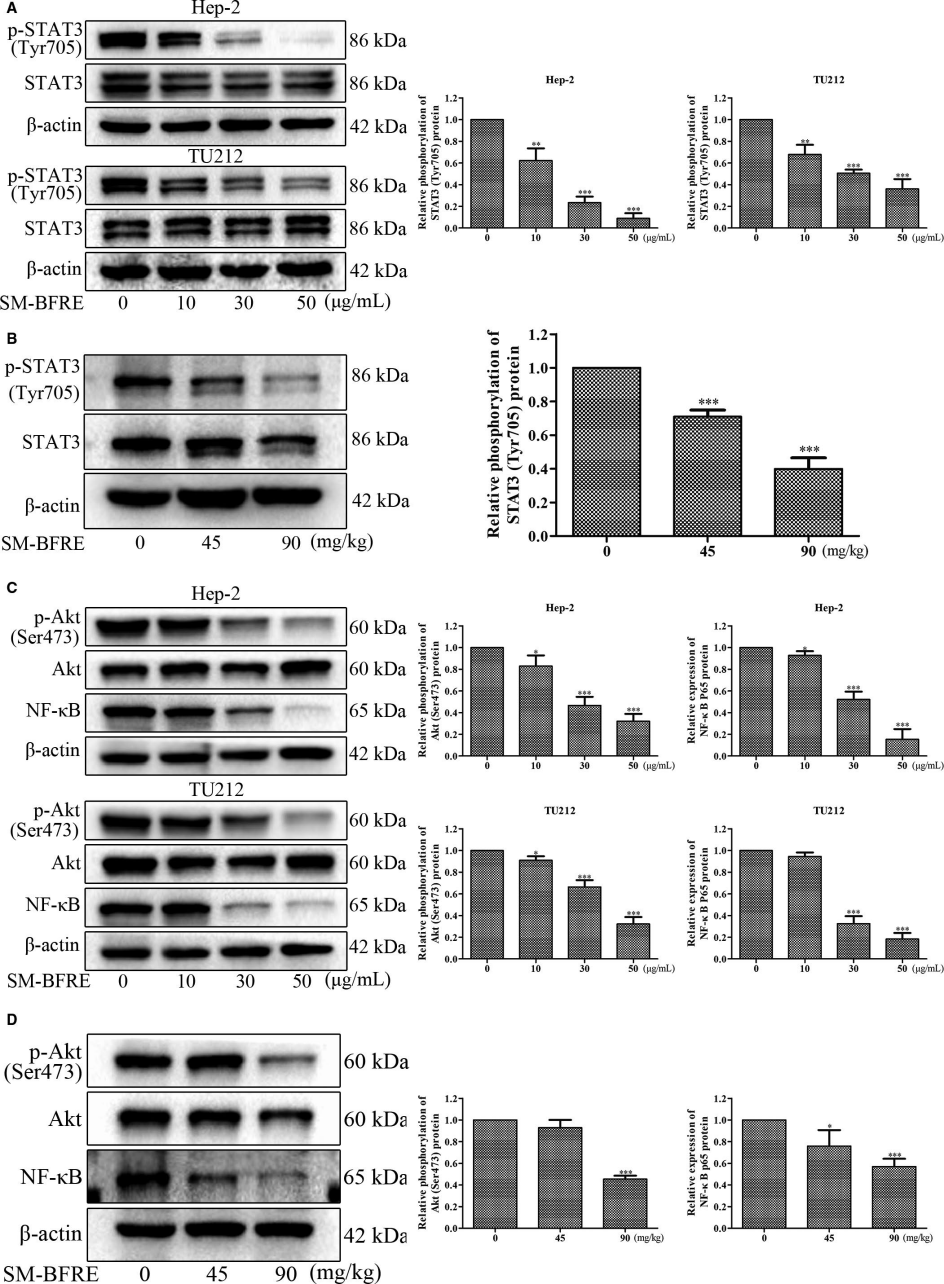
Regulation of STAT3 and Akt/NF‐κB signalling pathway by SM‐BFRE in vitro and in vivo. (A) The relative expression of p‐STAT3 (Tyr705) was detected by Western blot in two laryngeal carcinoma cells incubated with SM‐BFRE (0, 10, 30 and 50 μg/mL) for 24 h. (B) Proteins were extracted from transplanted tumour tissues, and the relative expression of p‐STAT3 (Tyr705) was detected by Western blot analysis. (C) The relative expression of p‐Akt (Ser473) and NF‐κB was detected by Western blot in two laryngeal carcinoma cells incubated with SM‐BFRE (0, 10, 30 and 50 μg/mL) for 24 h. (D) Proteins were extracted from transplanted tumour tissues, and the relative expression of p‐Akt (Ser473) and NF‐κB was detected by Western blot analysis. β‐actin was used as a control. *P < .05, **P < .01 or ***P < .001 compare to control (0 μg/mL or 0 mg/kg served as control)

Signal transduction and activator of transcription 3 (STAT3) is highly phosphorylated in almost all head and neck tumours.^21^ Studies have shown that STAT3 can regulate the genes that inhibit the apoptotic pathway and ultimately contribute to the development of tumours.^22^ In vitro and in vivo Western blot analysis showed that SM‐BFRE dose‐dependently inhibited the expression of p‐STAT3 (Tyr705), but did not inhibit the protein level of total STAT3 (Figure 6A, B). This suggested that SM‐BFRE might induce the apoptosis of laryngeal cancer cells in vitro and in vivo through the inhibition of STAT3 signalling.

In cancer cells, nuclear factor κB (NF‐κB) can be regulated by the PI3K/Akt signalling pathway and eventually enters the nucleus and inhibits tumour cells apoptosis.^23^ Western blot assay in vitro and in vivo results showed that SM‐BFRE dose‐dependently inhibited the expression of NF‐κB and p‐Akt (Ser473), but did not inhibit the protein level of total Akt (Figure 6C, D). This suggested that SM‐BFRE induced the apoptosis of laryngeal cancer cells through the inhibition of Akt/ NF‐κB signalling in vitro and in vivo.

## 4 DISCUSSION

Laryngeal carcinoma is a common type of tumour in the respiratory system. In the past 40 years, the five‐year survival ratio of sufferers has decreased from 66% to 63%.^24^ However, the current treatment of laryngeal cancer makes its prognosis unsatisfactory and there is an urgent need for finding more effective and feasible methods to treat laryngeal cancer.

Increasingly, studies have shown that natural products derived from plants play a crucial role in the treatment of malignant tumours.^25^ Such natural products have the advantage of killing cancer cells directly, in addition to less toxicity and side effects, and prolonging the life span of patients.^26^

Biflavonoids have been found to possess extensive pharmacological activity and are the main material basis of the anti‐tumour effect of Selaginella plants.^27^, ^28^ The present research indicated that SM‐BFRE suppressed the multiplication of laryngeal carcinoma cells in a dose‐ and time‐dependent manner. However, it had no obvious toxic effects on normal laryngeal epithelial cells (Figure 2).

Metastasis of cancer cells is a critical step in the development of most malignant tumours, which represents a poor prognosis for patients and leads to a more advanced stage of cancer.^29^ Tumour cell migration and invasion are the initial stages of carcinoma metastasis.^30^ Through the scratch assay, treatment with SM‐BFRE significantly reduced the wound healing ability of laryngeal carcinoma cell lines in a dose‐ and time‐dependent manner (Figure 3), suggesting that the inhibitory ability of SM‐BFRE for the metastasis of laryngeal cancer was positive and obvious.

Programmed cell death (PCD), commonly known as apoptosis, is of vital importance in clearing damaged cells and maintaining cellular homeostasis, and its disorders may lead to cancer.^31^ Apoptosis is accompanied by some typical features, such as cell shrinkage and floating, nuclear fragmentation, chromatin condensation and production of apoptotic bodies.^32^, ^33^ In this study, Hoechst 33 258 and Annexin V‐FITC/PI double staining assay indicated that SM‐BFRE treatment dose‐dependently resulted in the apoptosis of laryngeal carcinoma cells (Figure 4A‐C).

The two basic apoptotic signalling pathways are the extrinsic and intrinsic pathways.^29^ Activation of caspase by cell surface death receptors initiates the extrinsic pathway in response to homologous death ligands.^34^ The intrinsic apoptotic pathway (the mitochondrial pathway) is regulated by Bcl‐2 protein family (including anti‐apoptotic protein Bcl‐2 and pro‐apoptotic protein Bax).^32^, ^35^ A ratio of Bcl‐2/Bax was usually use as the basis for determining apoptosis, and an increased ratio represents anti‐cell apoptosis.^36^ The Bcl‐2 family controls the release of cytochrome c (Cyt‐c) by modulating the mitochondrial membrane permeability.^29^ Caspases are a group of proteases that have a similar structure in the cytoplasm. Caspase is responsible for the selective cleavage of certain proteins, and the result of cleavage is to activate or inactivate the target protein rather than completely degrade it. Under the action of apoptosis‐inducing factors, Cyt‐c in mitochondria is released into the cytoplasm and forms apoptotic bodies with caspase‐9 precursors, resulting in the excision of pro caspase‐9 in an active form. The activated cleaved caspase‐9 further cleaves the downstream caspase‐3 zymogen molecule to activate it and eventually induces apoptosis in the cells.^37^, ^38^ Poly ADP‐ribose polymerase (PARP) acts as a target protein downstream of caspase‐3 and affects the repair of cancer cells. Activated caspase‐3 prevents DNA repair by cleaving PARP. In the present study, Western blot assay in vitro indicated that SM‐BFRE might activate the mitochondrial pathway to induce apoptosis in laryngeal carcinoma cell lines (Figure 4D). Furthermore, Hep‐2‐bearing nude mouse models were established to investigate the pro‐apoptotic effects of SM‐BFRE in vivo. After treatment, SM‐BFRE dose‐dependently inhibited the growth of transplanted tumours in nude mice and exerted pro‐apoptotic effect by activating the mitochondrial apoptotic pathway in vivo (Figure 5).

Most anticancer drugs known today act by either activating or inhibiting multiple signalling pathways. In recent years, STAT3 has become an emerging target in cancer treatment.^39^ STAT3, a transcription factor, regulates the expression of genes involved in the cell cycle, cell survival and immune response associated with multiple carcinoma progression and malignant tumours.^40^ Once activated by phosphorylation, STAT3 forms a homodimer and translocates to the nucleus where it binds to DNA and promotes the translation of target genes associated with anti‐apoptosis.^41^ Phosphorylation of STAT3 at Tyr705 site (p‐STAT3 (Tyr705)) site is generally considered to be a prerequisite for the activation of the STAT3 signalling pathway. Besides the mitochondrial apoptotic pathway, Western blot analysis indicated that SM‐BFRE might also exert its anti‐laryngeal cancer effect by inhibiting the STAT3 signalling pathway (Figure 6A, B).

NF‐κB is a closely related family of transcription factors and an important nuclear transcription factor in cells. It is mainly involved in the body's inflammatory response and immune response. However, a growing number of reports have indicated that NF‐κB modulates the expression of genes that are of vital importance in the development and progression of tumours, including cell proliferation, migration and apoptosis.^42^ It is generally believed that NF‐κB exists in the cytoplasm in the form of a dimer in combination with its inhibitor (IκBs) in an inactive state. When stimulated, the IκB protein is degraded by phosphorylation and ubiquitination, and the NF‐κB dimer is released and finally transferred to the nucleus, which binds to the target gene to promote its transcription. Studies have indicated that the activation of the PI3K/Akt signalling pathway is also involved in the activation of NF‐κB.^42^ Akt has two phosphorylation sites (Thr308 and Ser473) that are activated by dual phosphorylation. Phosphorylation of the Thr308 activates the Akt moiety, but the entirety of the Akt functional activity requires phosphorylation of Ser473.^43^ The decrease in phosphorylated Akt (p‐Akt Ser473) expression is a key indicator of the activation of PI3K/Akt pathway. As mentioned earlier, NF‐κB has a regulatory effect on apoptosis, and NF‐κB is generally thought to inhibit cell apoptosis. Both in vitro and in vivo results were consistent with our conjecture that SM‐BFRE‐induced apoptosis in laryngeal carcinoma cells might be attributed to the inhibition of the Akt/NF‐κB signalling pathway (Figure 6C, D).

**FIGURE 7:**
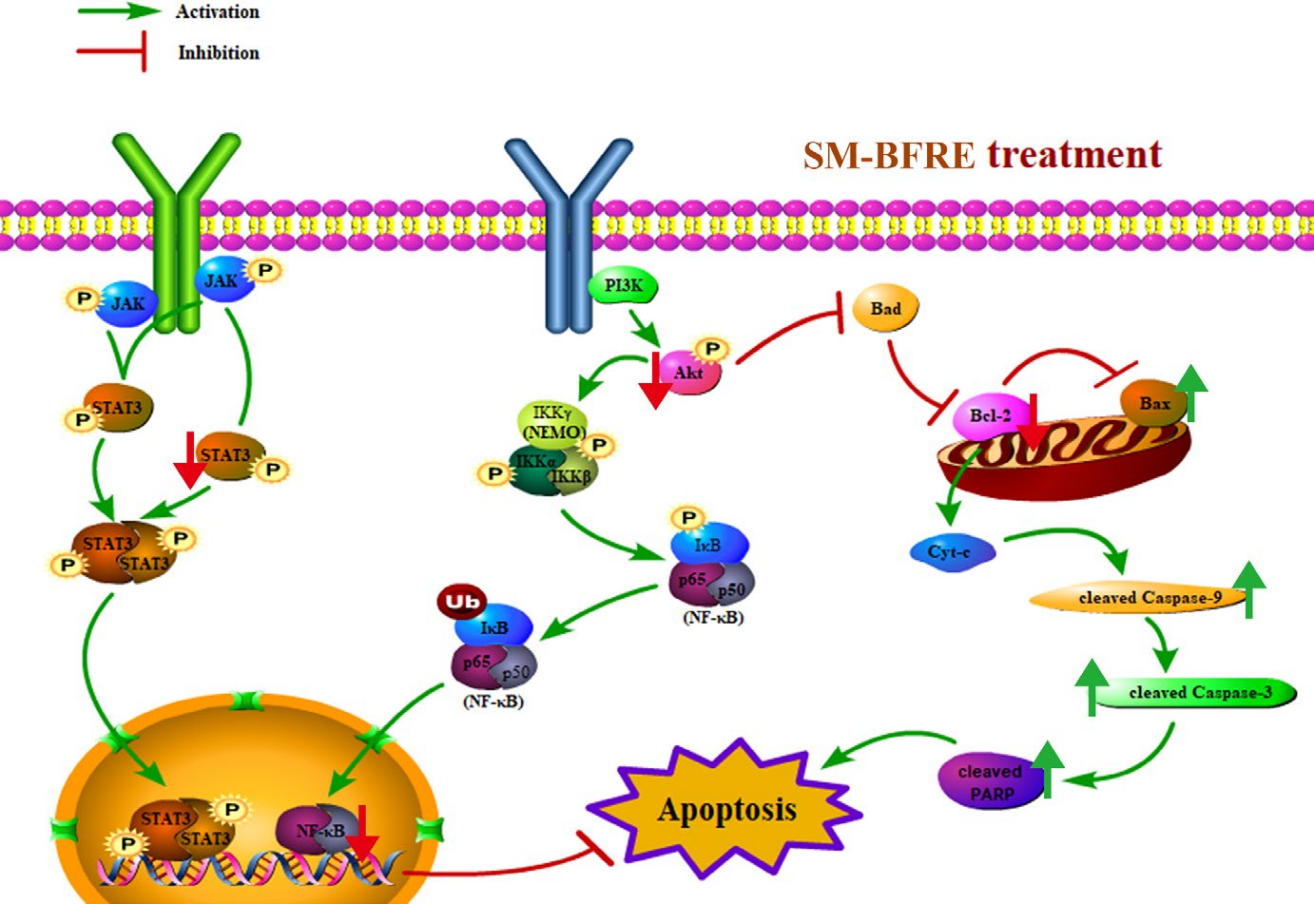
A schematic summary for the mechanisms of SM‐BFRE‐induced laryngeal carcinoma cells apoptosis in the present study

In summary, HPLC analysis revealed that SM‐BFRE contains a large amount of biflavonoids that might play a major role in the anti‐laryngeal cancer effects of SM‐BFRE. Pharmacological studies showed that SM‐BFRE significantly inhibited the proliferation and migration of laryngeal cancer cell lines with no obvious toxicity towards normal laryngeal epithelial cells. Experiments conducted in vitro and in vivo demonstrated that SM‐BFRE could induce apoptosis in laryngeal carcinoma cells, and further verified that its anti‐laryngeal cancer effect that involved activation of the mitochondrial apoptotic pathway and inhibition of the STAT3 and Akt/NF‐κB signalling pathways (Figure 7). The results of our current study suggest that SM‐BFRE can be considered as a potential chemotherapeutic drug for laryngeal carcinoma.

## CONFLICT OF INTEREST

The authors confirm that there are no conflicts of interest.

## AUTHOR CONTRIBUTION

**Huiqi Huang:** Conceptualization (lead); Data curation (lead); Formal analysis (lead); Methodology (lead); Project administration (equal); Software (lead); Validation (lead); Visualization (lead); Writing‐original draft (lead); Writing‐review & editing (lead). **Ji Hao:** Formal analysis (supporting); Investigation (supporting); Software (supporting); Visualization (supporting). **Kejian Pang:** Formal analysis (supporting); Investigation (supporting); Resources (supporting). **Yibing Lv:** Formal analysis (supporting); Project administration (equal). **Dingrong Wan:** Investigation (supporting); Resources (lead). **Chaoqun Wu:** Project administration (equal). **Yuanren Ma:** Investigation (supporting). **Xinzhou Yang:** Conceptualization (lead); Funding acquisition (lead); Project administration (lead); Resources (lead); Supervision (lead); Writing‐review & editing (lead). **Wei Kevin Zhang:** Conceptualization (lead); Methodology (lead); Project administration (lead); Supervision (lead); Writing‐review & editing (supporting).

## Supporting information

Supplementary Material

## Data Availability

The data that support the findings of this study are available from the corresponding author upon reasonable request.
