# Supplementary material for: A biflavonoid‐rich extract from Selaginella moellendorffii Hieron. induces apoptosis via STAT3 and Akt/NF‐κB signalling pathways in laryngeal carcinoma
Source: J Cell Mol Med. 2020 Sep 1;24(20):11922–35. doi: 10.1111/jcmm.15812 (PMC7579697; doi:10.1111/jcmm.15812)
Supplement: Supplementary file 1 — Supplementary Material [file JCMM-24-11922-s001.docx]

***Supplementary Material***

**Figure S1.** The specimen of *Selaginella moellendorfii* Hieron.


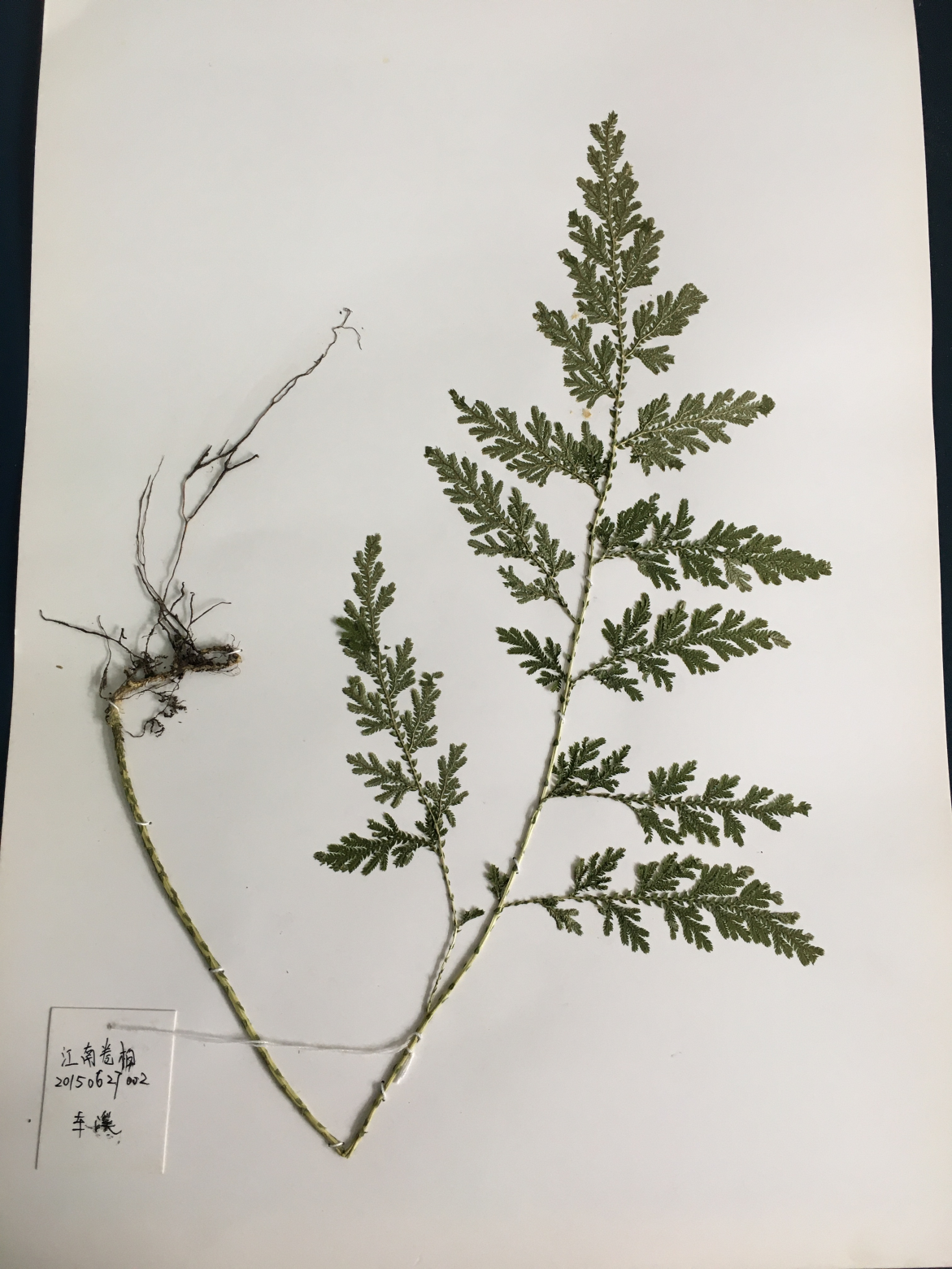


**Figure S2**. The cell viability and IC_50_ values of two laryngeal cancer cells after treatment with 6 pure compounds for 12, 24 and 48 h.


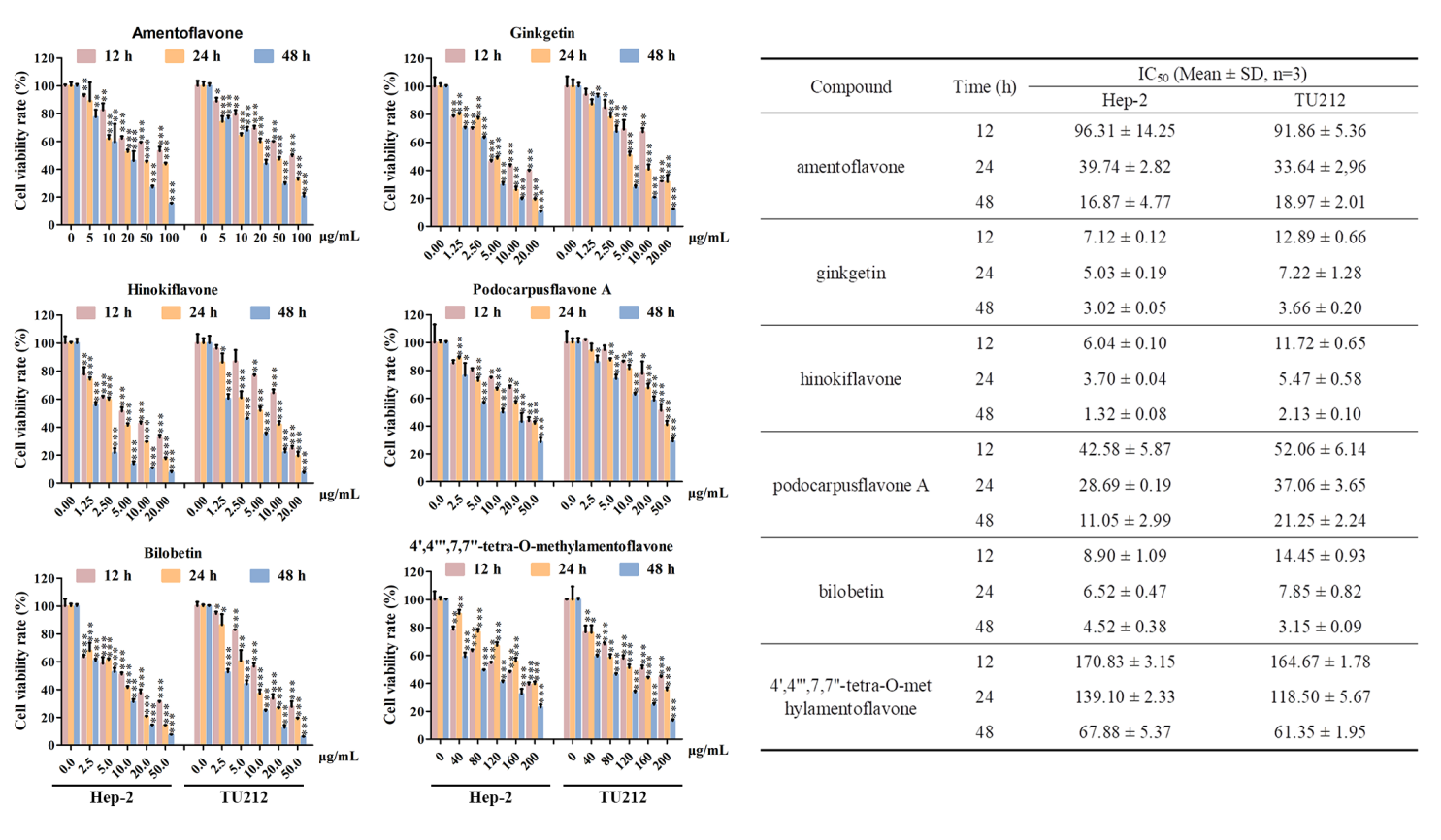


**Table S1.** The cell inhibitory rate of two laryngeal cancer cells after treatment with 100 μg/mL SM-BFRE, the mixture of major compounds and the mixture of minor compounds for 24 h.

| Compound (100 μg/mL) | Hep-2 | TU212 |
| --- | --- | --- |
| SM-BFRE | 75.20 ± 1.14 | 68.60 ± 1.22 |
| The mixture of major compounds | 71.57 ± 2.35 | 64.43 ± 1.27 |
| The mixture of minor compounds | 2.44 ± 0.96 | 3.61 ± 1.36 |
